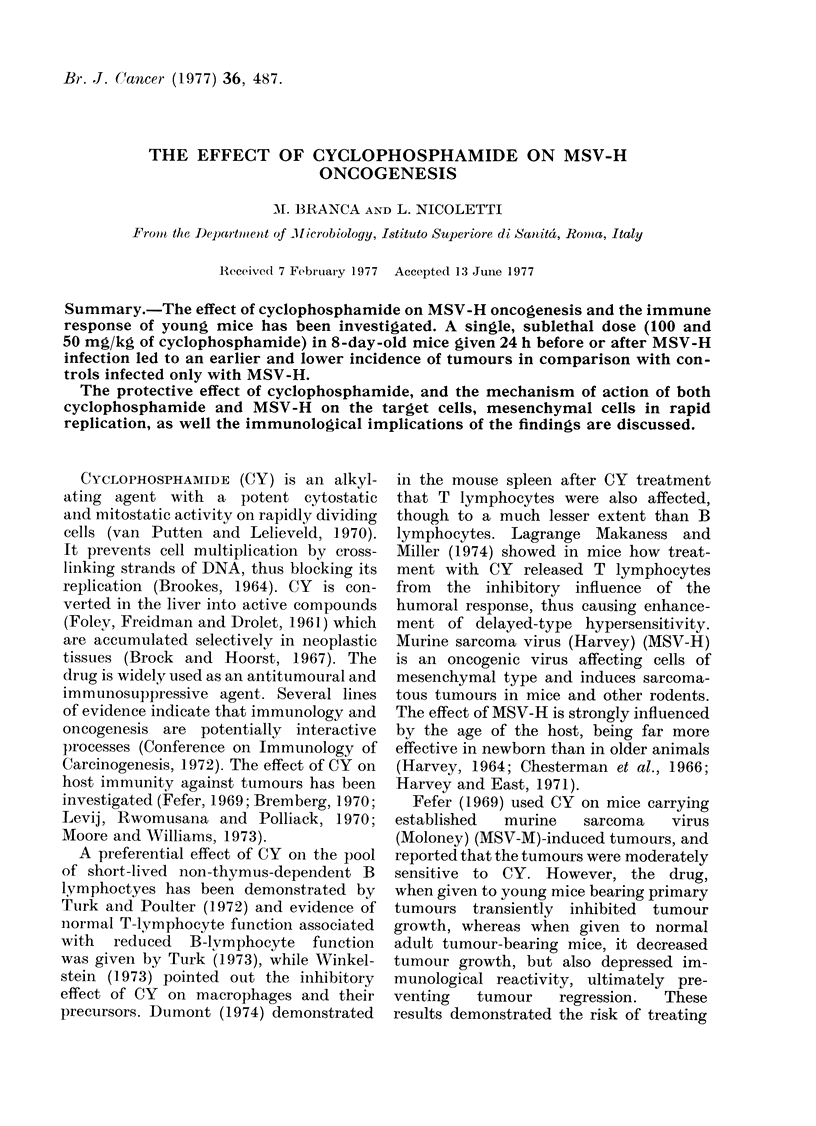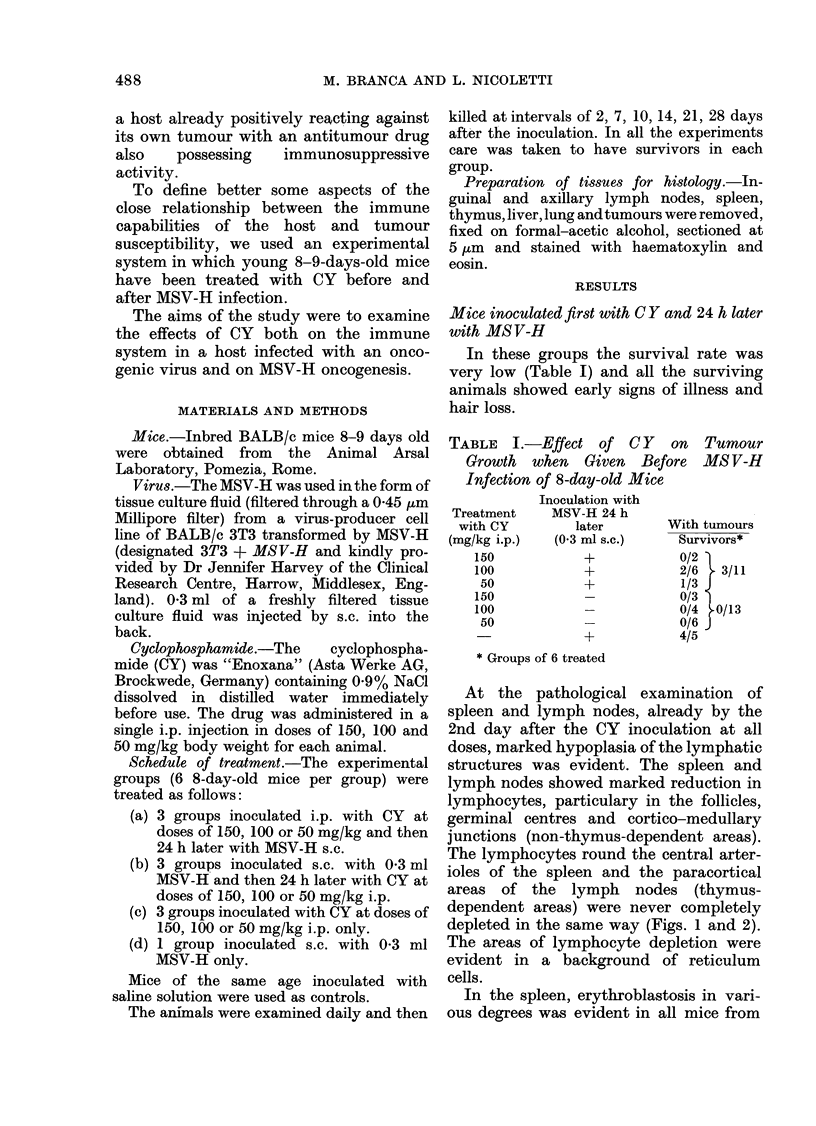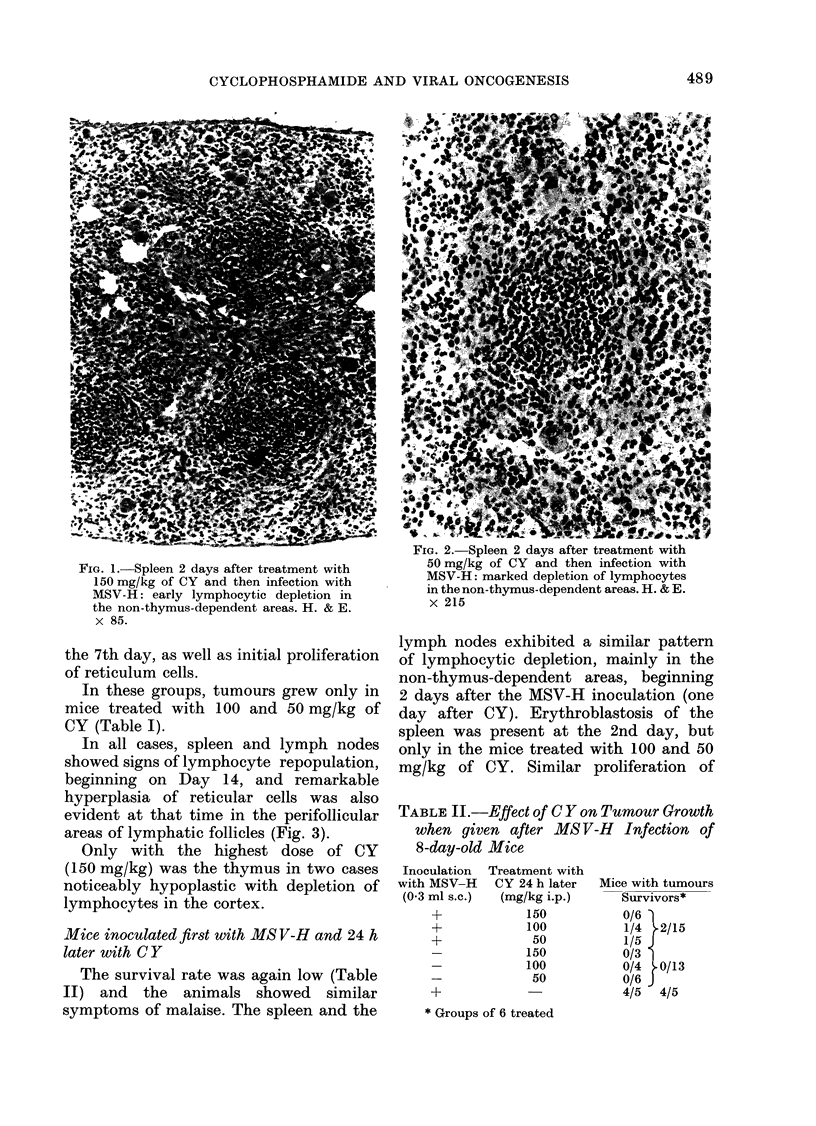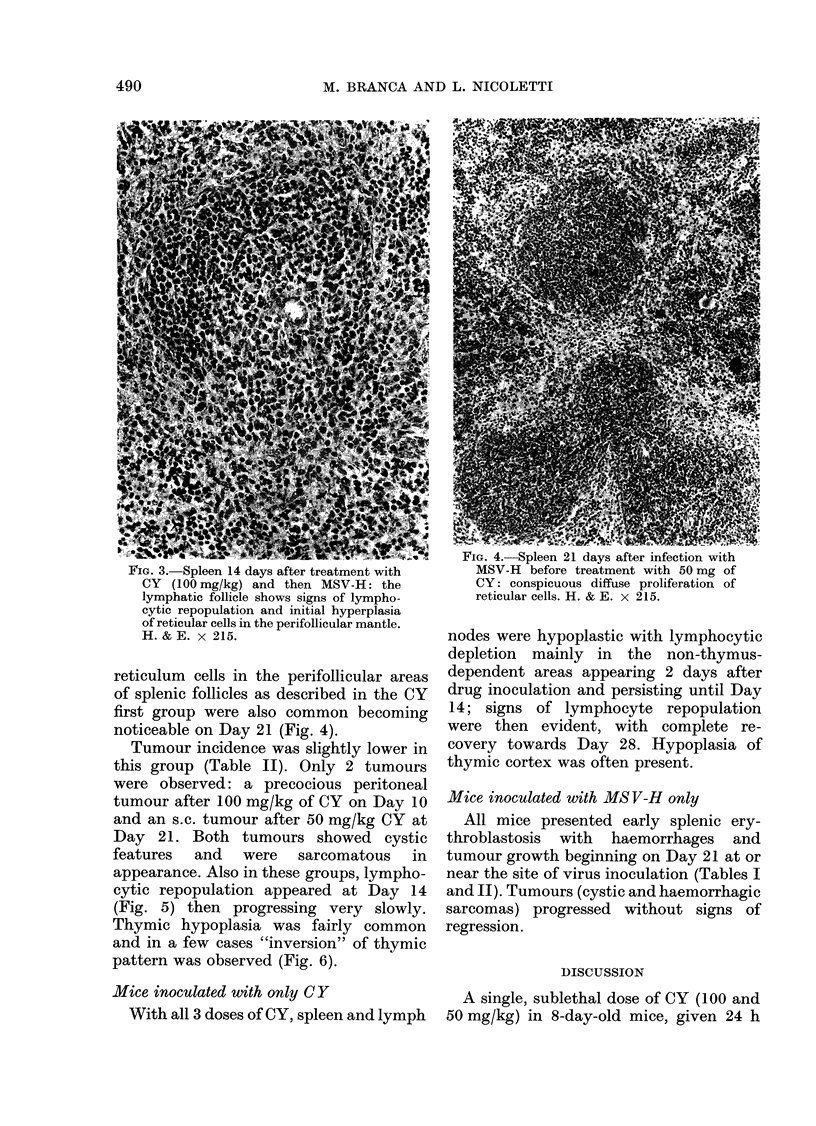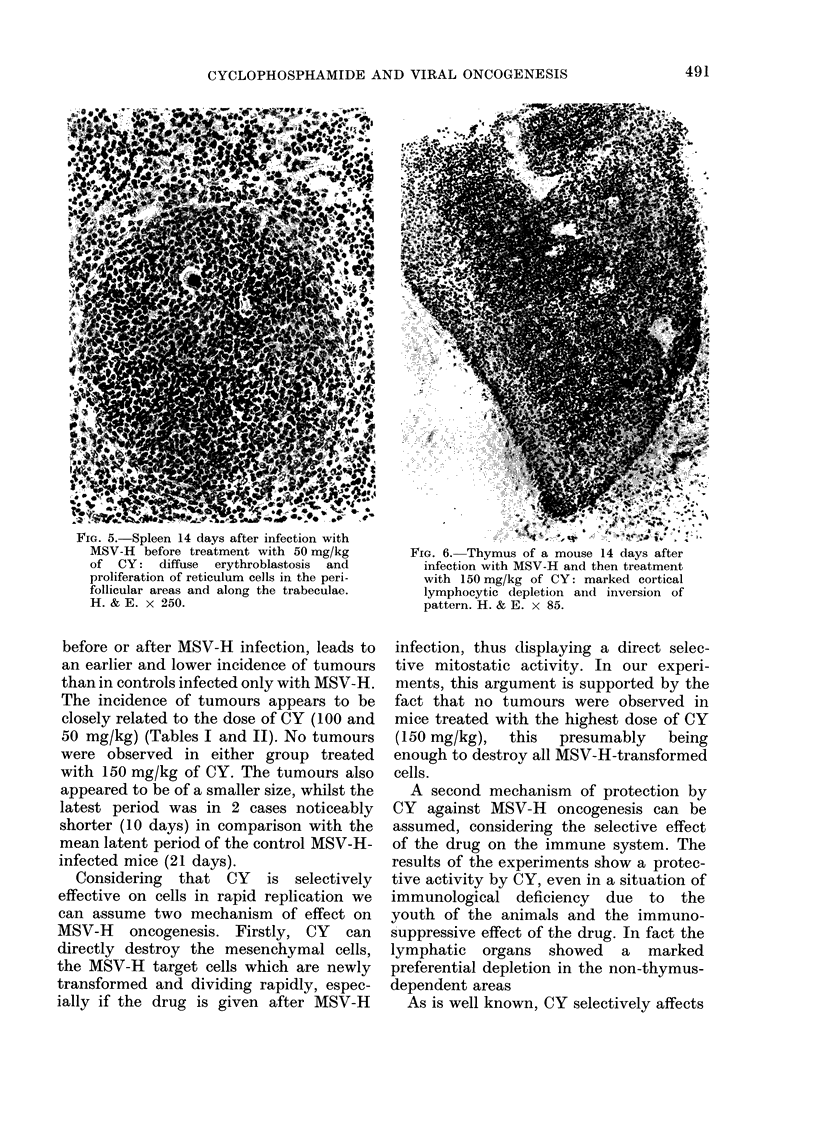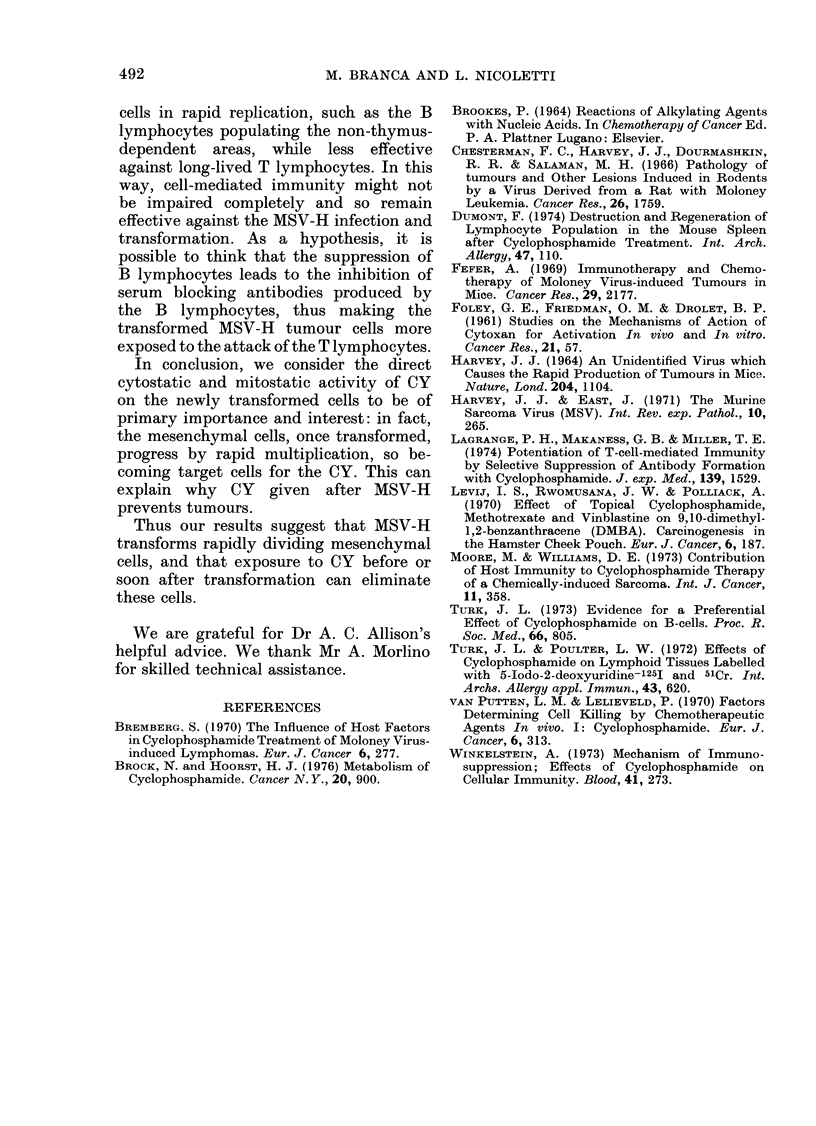# The effect of cyclophosphamide on MSV-H oncogenesis.

**DOI:** 10.1038/bjc.1977.218

**Published:** 1977-10

**Authors:** M. Branca, L. Nicoletti

## Abstract

**Images:**


Br. J. Cancer (1977) 36, 487.

### THE EFFECT OF CYCLOPHOSPHAMIDE ON MSV-H ONCOGENESIS

M. BRANCA AND L. NICOLETTI

*From the Department of Microbiology, Istituto Superiore di Sanitá, Roma, Italy*

Received 7 February 1977 Accepted 13 June 1977

**Summary.—The effect of cyclophosphamide on MSV-H oncogenesis and the immune response of young mice has been investigated. A single, sublethal dose (100 and 50 mg/kg of cyclophosphamide) in 8-day-old mice given 24 h before or after MSV-H infection led to an earlier and lower incidence of tumours in comparison with controls infected only with MSV-H.**

**The protective effect of cyclophosphamide, and the mechanism of action of both cyclophosphamide and MSV-H on the target cells, mesenchymal cells in rapid replication, as well the immunological implications of the findings are discussed.**

CYCLOPHOSPHAMIDE (CY) is an alkylating agent with a potent cytostatic and mitostatic activity on rapidly dividing cells (van Putten and Lelieveld, 1970). It prevents cell multiplication by cross-linking strands of DNA, thus blocking its replication (Brookes, 1964). CY is converted in the liver into active compounds (Foley, Freidman and Drolet, 1961) which are accumulated selectively in neoplastic tissues (Brock and Hoorst, 1967). The drug is widely used as an antitumoural and immunosuppressive agent. Several lines of evidence indicate that immunology and oncogenesis are potentially interactive processes (Conference on Immunology of Carcinogenesis, 1972). The effect of CY on host immunity against tumours has been investigated (Fefer, 1969; Bremberg, 1970; Levij, Rwomusana and Polliack, 1970; Moore and Williams, 1973).

A preferential effect of CY on the pool of short-lived non-thymus-dependent B lymphoctyes has been demonstrated by Turk and Poulter (1972) and evidence of normal T-lymphocyte function associated with reduced B-lymphocyte function was given by Turk (1973), while Winkelstein (1973) pointed out the inhibitory effect of CY on macrophages and their precursors. Dumont (1974) demonstrated in the mouse spleen after CY treatment that T lymphocytes were also affected, though to a much lesser extent than B lymphocytes. Lagrange Makaness and Miller (1974) showed in mice how treatment with CY released T lymphocytes from the inhibitory influence of the humoral response, thus causing enhancement of delayed-type hypersensitivity. Murine sarcoma virus (Harvey) (MSV-H) is an oncogenic virus affecting cells of mesenchymal type and induces sarcomatous tumours in mice and other rodents. The effect of MSV-H is strongly influenced by the age of the host, being far more effective in newborn than in older animals (Harvey, 1964; Chesterman *et al.*, 1966; Harvey and East, 1971).

Fefer (1969) used CY on mice carrying established murine sarcoma virus (Moloney) (MSV-M)-induced tumours, and reported that the tumours were moderately sensitive to CY. However, the drug, when given to young mice bearing primary tumours transiently inhibited tumour growth, whereas when given to normal adult tumour-bearing mice, it decreased tumour growth, but also depressed immunological reactivity, ultimately preventing tumour regression. These results demonstrated the risk of treating a host already positively reacting against its own tumour with an antitumour drug also possessing immunosuppressive activity.

To define better some aspects of the close relationship between the immune capabilities of the host and tumour susceptibility, we used an experimental system in which young 8–9-days-old mice have been treated with CY before and after MSV-H infection.

The aims of the study were to examine the effects of CY both on the immune system in a host infected with an oncogenic virus and on MSV-H oncogenesis.

### MATERIALS AND METHODS

*Mice.*—Inbred BALB/c mice 8–9 days old were obtained from the Animal Arsal Laboratory, Pomezia, Rome.

*Virus.*—The MSV-H was used in the form of tissue culture fluid (filtered through a 0·45 $\mu$m Millipore filter) from a virus-producer cell line of BALB/c 3T3 transformed by MSV-H (designated *3T3 + MSV-H* and kindly provided by Dr Jennifer Harvey of the Clinical Research Centre, Harrow, Middlesex, England). 0·3 ml of a freshly filtered tissue culture fluid was injected by s.c. into the back.

*Cyclophosphamide.*—The cyclophosphamide (CY) was "Enoxana" (Asta Werke AG, Brockwede, Germany) containing 0·9% NaCl dissolved in distilled water immediately before use. The drug was administered in a single i.p. injection in doses of 150, 100 and 50 mg/kg body weight for each animal.

*Schedule of treatment.*—The experimental groups (6 8-day-old mice per group) were treated as follows:

(a) 3 groups inoculated i.p. with CY at doses of 150, 100 or 50 mg/kg and then 24 h later with MSV-H s.c.
(b) 3 groups inoculated s.c. with 0·3 ml MSV-H and then 24 h later with CY at doses of 150, 100 or 50 mg/kg i.p.
(c) 3 groups inoculated with CY at doses of 150, 100 or 50 mg/kg i.p. only.
(d) 1 group inoculated s.c. with 0·3 ml MSV-H only.

Mice of the same age inoculated with saline solution were used as controls.

The animals were examined daily and then killed at intervals of 2, 7, 10, 14, 21, 28 days after the inoculation. In all the experiments care was taken to have survivors in each group.

*Preparation of tissues for histology.*—Inguinal and axillary lymph nodes, spleen, thymus, liver, lung and tumours were removed, fixed on formal–acetic alcohol, sectioned at 5 $\mu$m and stained with haematoxylin and eosin.

### RESULTS

### *Mice inoculated first with CY and 24 h later with MSV-H*

In these groups the survival rate was very low (Table I) and all the surviving animals showed early signs of illness and hair loss.

TABLE I.—*Effect of CY on Tumour Growth when Given Before MSV-H Infection of 8-day-old Mice*

| Treatment with CY (mg/kg i.p.) | Inoculation with MSV-H 24 h later (0·3 ml s.c.) | With tumours / Survivors* | |
|---|---|---|---|
| 150 | + | 0/2 | 3/11 |
| 100 | + | 2/6 | |
| 50 | + | 1/3 | |
| 150 | − | 0/3 | 0/13 |
| 100 | − | 0/4 | |
| 50 | − | 0/6 | |
| — | + | 4/5 | |

* Groups of 6 treated

At the pathological examination of spleen and lymph nodes, already by the 2nd day after the CY inoculation at all doses, marked hypoplasia of the lymphatic structures was evident. The spleen and lymph nodes showed marked reduction in lymphocytes, particulary in the follicles, germinal centres and cortico–medullary junctions (non-thymus-dependent areas). The lymphocytes round the central arterioles of the spleen and the paracortical areas of the lymph nodes (thymus-dependent areas) were never completely depleted in the same way (Figs. 1 and 2). The areas of lymphocyte depletion were evident in a background of reticulum cells.

In the spleen, erythroblastosis in various degrees was evident in all mice from

FIG. 1.—Spleen 2 days after treatment with 150 mg/kg of CY and then infection with MSV-H: early lymphocytic depletion in the non-thymus-dependent areas. H. & E. × 85.

FIG. 2.—Spleen 2 days after treatment with 50 mg/kg of CY and then infection with MSV-H: marked depletion of lymphocytes in the non-thymus-dependent areas. H. & E. × 215

the 7th day, as well as initial proliferation of reticulum cells.

In these groups, tumours grew only in mice treated with 100 and 50 mg/kg of CY (Table I).

In all cases, spleen and lymph nodes showed signs of lymphocyte repopulation, beginning on Day 14, and remarkable hyperplasia of reticular cells was also evident at that time in the perifollicular areas of lymphatic follicles (Fig. 3).

Only with the highest dose of CY (150 mg/kg) was the thymus in two cases noticeably hypoplastic with depletion of lymphocytes in the cortex.

*Mice inoculated first with MSV-H and 24 h later with CY*

The survival rate was again low (Table II) and the animals showed similar symptoms of malaise. The spleen and the lymph nodes exhibited a similar pattern of lymphocytic depletion, mainly in the non-thymus-dependent areas, beginning 2 days after the MSV-H inoculation (one day after CY). Erythroblastosis of the spleen was present at the 2nd day, but only in the mice treated with 100 and 50 mg/kg of CY. Similar proliferation of

TABLE II.—*Effect of CY on Tumour Growth when given after MSV-H Infection of 8-day-old Mice*

| Inoculation with MSV–H (0·3 ml s.c.) | Treatment with CY 24 h later (mg/kg i.p.) | Mice with tumours / Survivors* | |
|---|---|---|---|
| + | 150 | 0/6 | 2/15 |
| + | 100 | 1/4 | |
| + | 50 | 1/5 | |
| – | 150 | 0/3 | 0/13 |
| – | 100 | 0/4 | |
| – | 50 | 0/6 | |
| + | — | 4/5 | 4/5 |

* Groups of 6 treated

FIG. 3.—Spleen 14 days after treatment with CY (100 mg/kg) and then MSV-H: the lymphatic follicle shows signs of lymphocytic repopulation and initial hyperplasia of reticular cells in the perifollicular mantle. H. & E. × 215.

FIG. 4.—Spleen 21 days after infection with MSV-H before treatment with 50 mg of CY: conspicuous diffuse proliferation of reticular cells. H. & E. × 215.

reticulum cells in the perifollicular areas of splenic follicles as described in the CY first group were also common becoming noticeable on Day 21 (Fig. 4).

Tumour incidence was slightly lower in this group (Table II). Only 2 tumours were observed: a precocious peritoneal tumour after 100 mg/kg of CY on Day 10 and an s.c. tumour after 50 mg/kg CY at Day 21. Both tumours showed cystic features and were sarcomatous in appearance. Also in these groups, lymphocytic repopulation appeared at Day 14 (Fig. 5) then progressing very slowly. Thymic hypoplasia was fairly common and in a few cases "inversion" of thymic pattern was observed (Fig. 6).

*Mice inoculated with only CY*

With all 3 doses of CY, spleen and lymph nodes were hypoplastic with lymphocytic depletion mainly in the non-thymus-dependent areas appearing 2 days after drug inoculation and persisting until Day 14; signs of lymphocyte repopulation were then evident, with complete recovery towards Day 28. Hypoplasia of thymic cortex was often present.

*Mice inoculated with MSV-H only*

All mice presented early splenic erythroblastosis with haemorrhages and tumour growth beginning on Day 21 at or near the site of virus inoculation (Tables I and II). Tumours (cystic and haemorrhagic sarcomas) progressed without signs of regression.

### DISCUSSION

A single, sublethal dose of CY (100 and 50 mg/kg) in 8-day-old mice, given 24 h

FIG. 5.—Spleen 14 days after infection with MSV-H before treatment with 50 mg/kg of CY: diffuse erythroblastosis and proliferation of reticulum cells in the perifollicular areas and along the trabeculae. H. & E. × 250.

FIG. 6.—Thymus of a mouse 14 days after infection with MSV-H and then treatment with 150 mg/kg of CY: marked cortical lymphocytic depletion and inversion of pattern. H. & E. × 85.

before or after MSV-H infection, leads to an earlier and lower incidence of tumours than in controls infected only with MSV-H. The incidence of tumours appears to be closely related to the dose of CY (100 and 50 mg/kg) (Tables I and II). No tumours were observed in either group treated with 150 mg/kg of CY. The tumours also appeared to be of a smaller size, whilst the latest period was in 2 cases noticeably shorter (10 days) in comparison with the mean latent period of the control MSV-H-infected mice (21 days).

Considering that CY is selectively effective on cells in rapid replication we can assume two mechanism of effect on MSV-H oncogenesis. Firstly, CY can directly destroy the mesenchymal cells, the MSV-H target cells which are newly transformed and dividing rapidly, especially if the drug is given after MSV-H infection, thus displaying a direct selective mitostatic activity. In our experiments, this argument is supported by the fact that no tumours were observed in mice treated with the highest dose of CY (150 mg/kg), this presumably being enough to destroy all MSV-H-transformed cells.

A second mechanism of protection by CY against MSV-H oncogenesis can be assumed, considering the selective effect of the drug on the immune system. The results of the experiments show a protective activity by CY, even in a situation of immunological deficiency due to the youth of the animals and the immunosuppressive effect of the drug. In fact the lymphatic organs showed a marked preferential depletion in the non-thymus-dependent areas

As is well known, CY selectively affects cells in rapid replication, such as the B lymphocytes populating the non-thymus-dependent areas, while less effective against long-lived T lymphocytes. In this way, cell-mediated immunity might not be impaired completely and so remain effective against the MSV-H infection and transformation. As a hypothesis, it is possible to think that the suppression of B lymphocytes leads to the inhibition of serum blocking antibodies produced by the B lymphocytes, thus making the transformed MSV-H tumour cells more exposed to the attack of the T lymphocytes.

In conclusion, we consider the direct cytostatic and mitostatic activity of CY on the newly transformed cells to be of primary importance and interest: in fact, the mesenchymal cells, once transformed, progress by rapid multiplication, so becoming target cells for the CY. This can explain why CY given after MSV-H prevents tumours.

Thus our results suggest that MSV-H transforms rapidly dividing mesenchymal cells, and that exposure to CY before or soon after transformation can eliminate these cells.

We are grateful for Dr A. C. Allison's helpful advice. We thank Mr A. Morlino for skilled technical assistance.

### REFERENCES

Bremberg, S. (1970) The Influence of Host Factors in Cyclophosphamide Treatment of Moloney Virus-induced Lymphomas. *Eur. J. Cancer* **6**, 277.

Brock, N. and Hoorst, H. J. (1976) Metabolism of Cyclophosphamide. *Cancer N.Y.*, **20**, 900.

Brookes, P. (1964) Reactions of Alkylating Agents with Nucleic Acids. In *Chemotherapy of Cancer* Ed. P. A. Plattner Lugano: Elsevier.

Chesterman, F. C., Harvey, J. J., Dourmashkin, R. R. & Salaman, M. H. (1966) Pathology of tumours and Other Lesions Induced in Rodents by a Virus Derived from a Rat with Moloney Leukemia. *Cancer Res.*, **26**, 1759.

Dumont, F. (1974) Destruction and Regeneration of Lymphocyte Population in the Mouse Spleen after Cyclophosphamide Treatment. *Int. Arch. Allergy*, **47**, 110.

Fefer, A. (1969) Immunotherapy and Chemotherapy of Moloney Virus-induced Tumours in Mice. *Cancer Res.*, **29**, 2177.

Foley, G. E., Friedman, O. M. & Drolet, B. P. (1961) Studies on the Mechanisms of Action of Cytoxan for Activation *In vivo* and *In vitro*. *Cancer Res.*, **21**, 57.

Harvey, J. J. (1964) An Unidentified Virus which Causes the Rapid Production of Tumours in Mice. *Nature, Lond.* **204**, 1104.

Harvey, J. J. & East, J. (1971) The Murine Sarcoma Virus (MSV). *Int. Rev. exp. Pathol.*, **10**, 265.

Lagrange, P. H., Makaness, G. B. & Miller, T. E. (1974) Potentiation of T-cell-mediated Immunity by Selective Suppression of Antibody Formation with Cyclophosphamide. *J. exp. Med.*, **139**, 1529.

Levij, I. S., Rwomusana, J. W. & Polliack, A. (1970) Effect of Topical Cyclophosphamide, Methotrexate and Vinblastine on 9,10-dimethyl-1,2-benzanthracene (DMBA). Carcinogenesis in the Hamster Cheek Pouch. *Eur. J. Cancer*, **6**, 187.

Moore, M. & Williams, D. E. (1973) Contribution of Host Immunity to Cyclophosphamide Therapy of a Chemically-induced Sarcoma. *Int. J. Cancer*, **11**, 358.

Turk, J. L. (1973) Evidence for a Preferential Effect of Cyclophosphamide on B-cells. *Proc. R. Soc. Med.*, **66**, 805.

Turk, J. L. & Poulter, L. W. (1972) Effects of Cyclophosphamide on Lymphoid Tissues Labelled with 5-Iodo-2-deoxyuridine-$^{125}$I and $^{51}$Cr. *Int. Archs. Allergy appl. Immun.*, **43**, 620.

van Putten, L. M. & Lelieveld, P. (1970) Factors Determining Cell Killing by Chemotherapeutic Agents *In vivo*. I: Cyclophosphamide. *Eur. J. Cancer*, **6**, 313.

Winkelstein, A. (1973) Mechanism of Immunosuppression; Effects of Cyclophosphamide on Cellular Immunity. *Blood*, **41**, 273.